# Xiaoyao Pills Attenuate Inflammation and Nerve Injury Induced by Lipopolysaccharide in Hippocampal Neurons In Vitro

**DOI:** 10.1155/2020/8841332

**Published:** 2020-09-21

**Authors:** Yang Fang, Boyu Shi, Xiaobo Liu, Jie Luo, Zhili Rao, Rong Liu, Nan Zeng

**Affiliations:** Department of Pharmacology, College of Pharmacy, Chengdu University of TCM, Chengdu City, Sichuan Province 611137, China

## Abstract

Lipopolysaccharides (LPS) are proinflammation mediators that can induce the inflammatory model of the hippocampal neuron, and neuroinflammation participates in the pathophysiology of depression. Xiaoyao Pill is a classical Chinese medicine formula that has been used for the treatment of mental disorders such as depression in China since the Song dynasty. We established a hippocampal neuronal cell inflammation model by LPS and investigate the intervention effect and mechanism of Xiaoyao Pills. The expression levels of IL-6, TNF-*α*, IDO, 5-HT, brain-derived neurotrophic factor, and *β*-nerve growth factor were detected by enzyme-linked immunosorbent assay. mRNA levels of IL-6, TNF-*α*, 5-HT1A, IDO-1, brain-derived neurotrophic factor, nerve growth factor, tropomyosin receptor kinase B, tropomyosin receptor kinase A, and cAMP response element-binding protein were detected by reverse transcription-polymerase chain reaction. To further validate, protein expression was determined by western blot and immunofluorescence. Lipopolysaccharide-induced neuroinflammatory state resulted in the release of IL-6, TNF-*α*, and IDO and a decrease of BDNF, NGF, TrkB, TrkA, CREB, p-CREB, p-CREB/CREB, and SYP and inhibited hippocampal neurogenesis in the hippocampal neuron. Xiaoyao Pills significantly decreased the levels of IL-6, TNF-*α*, and IDO in cell supernatant and increased the expression of BDNF, NGF, TrkB, TrkA, CREB, p-CREB, p-CREB/CREB, and SYP as well as the average optical density of BrdU/NeuN double-labelled positive cells. Our study shows that lipopolysaccharides induce inflammation and nerve damage in hippocampal neurons, which are closely related to the pathological mechanism of depression. Xiaoyao Pills (XYW) play an important neuroprotective effect, which is related to its inhibition of neuronal inflammation and promoting the recovery of nerve injury. These results provide a pharmacologic basis for the treatment of depression of XYW in clinical application.

## 1. Introduction

Depression is a devastating psychiatric disease that prevails throughout the world and has profound effects on neural structure and function. It is characterized by psychophysiological changes, such as low mood, loss of self-feeling, sadness, irritability, and loss of interest in all activities [1]. The incidence of depression in every generation worldwide is increasing. Numerous studies have identified genetic factors, environmental factors, and stress as major risk factors for depression [2].

Although depression has been explained with many theories, such as the monoamine theory, hypothalamus–pituitary–adrenal (HPA) axis theory, neurotrophic hypothesis, and neuroinflammation theory, it is far more multifactorial. In a sense, inflammation is a static load involving the immune, endocrine, and nervous systems. Initially, the investigations focused mostly on the effects of systemic inflammation on the central nervous system (CNS). However, current research focuses on neuroinflammation that occurs within the CNS; these findings suggest that cytokine-mediated interventions may be valuable for treating depression in this population [3]. The process of neuroinflammation involves sentinel immune cells in the CNS, resident macrophages called microglia [4]. The activation of microglia could trigger neuroinflammation, further causing a variety of neuropsychiatric diseases, such as depression [5], Alzheimer's disease [6], and schizophrenia [7]. Neuroinflammation participates in the pathophysiology of depression by increasing proinflammatory cytokines, activating the hypothalamus–pituitary–adrenal axis, increasing glucocorticoid resistance, and affecting serotonin synthesis and metabolism, neuronal apoptosis and neurogenesis, and neuroplasticity [8]. The current research finds that proinflammatory cytokines inhibit the negative-feedback regulation of the HPA axis and cause the depletion of serotonin [9]. It also plays a key role in neuroendocrine, neurotransmitter depletion, neural plasticity, and local brain activity. Lipopolysaccharide (LPS) challenge could stimulate the acute inflammatory response that causes depressive symptoms in humans and rodents. Therefore, based on the close relationship between neuroinflammation and depression, LPS is often used to prepare depression-like models induced by inflammatory responses [10, 11].

Hippocampal atrophy is often observed in depressed patients and is considered a biomarker of depression risk [12]. The hippocampus is susceptible to neuroinflammatory. Numerous studies have shown that the proneurogenic effect of microglia cells is related to chronic neurodegeneration, and the activation of microglia cells plays a key role in inhibiting the hippocampus neurogenesis under stress and inflammatory conditions [13, 14]. Stress could also reduce neuronal dendrite branching and plasticity in the hippocampus neurons [15]. Hippocampal plasticity in depression involves hippocampal volume, hippocampal neurogenesis, and apoptosis of hippocampal neurons. Hippocampal neurogenesis in humans may be critical to the therapy of depression.

Xiaoyao San is included in the Chinese Pharmacopoeia *[*16*]*, which is a classical Chinese medicine formula. It is a prescription for Xiaoyao Pills. Xiaoyao San decoction comprises eight commonly used herbs *Bupleurum chinense* DC., *Angelica sinensis* Diels., *Paeonia lactiflora* Pall., *Atractylodes macrocephala* Koidz., *Mentha haplocalyx* Briq., *Poria cocos* Wolf., *Glycyrrhiza uralensis* Fisch., and *Zingiber officinale* Rosc. And if it is used for in vitro cell experiments directly, it will interfere with the experiment. Therefore, according to the concept of “serum pharmacology” proposed by Japanese scholar Hiroko Iwama, we used drug serum as the drug for in vitro experiments; it can reduce the interference of traditional Chinese medicine preparations on experiments in vitro, and it also meets the physiological process of pharmacological effects of Chinese medicine after being digested and absorbed by the body and biological metabolism [17]. At present, there have been many reports on the ingredients that may exist in the serum of Xiaoyao San: a total of 55 blood-containing components, including 16 original components, were identified in the rat-containing serum administered by the extracts of *Bupleurum chinense* DC. and *Paeonia lactiflora* Pall. The metabolic components are derived from saponin metabolites in *Bupleurum chinense* DC. and paeoniflorin metabolites in *Paeonia lactiflora* Pall [18].. The relative content of ligustilide was found in the drug-containing serum of *Angelica sinensis* Diels. And the extract is significantly higher than that in the original drug [19]. Metabolism components of 6 parent ingredients and 5 metabolic ingredients were found in the medicated serum of male rats administered with *Poria cocos* Wolf. extract [20]. Therefore, it is reliable to use drug serum for the study of Chinese medicine in vitro. Xiaoyao San has been used for the treatment of mental disorders such as depression for about nine hundred years in China since the Song dynasty. The antidepressant potential may be closely related to its pharmacological activity for invigorating the spleen, soothing the liver, nourishing the blood, and clearing away the liver fire due to blood deficiency [21]. Xiaoyao San improved the abnormalities of the tryptophan-kynurenine metabolic pathways in depressed rats and exerted antidepressant effects, changing biological indicators in rat hippocampus [22]. The modified Xiaoyao San can improve hippocampal neurogenesis by modulating cerebral oxygen-dependent fMRI signals in the brain of mice to play an antidepressant role [23]. Clinically, Xiaoyao Pills can improve symptoms of depression and enhance the quality of life in patients. It has a good therapeutic effect on depression and is worthy of clinical application [24, 25]. Previously, we found that Xiaoyao San was a highly effective formula to prevent depression by suppressing the HPA axis signal and improving the BDNF pathway signal in CUMS rats. However, whether Xiaoyao San can attenuate the release of proinflammatory cytokines, activate the BDNF signaling pathway, and promote neurogenesis during neuroinflammatory still remains unknown.

Our previous studies found that XYW ameliorated the depression-like behavior, decreased the levels of inflammatory indicators, increased those of neurotrophic factors and synaptic proteins, and restored Nissl bodies in acute stress mice and rat, thus improving depression in vivo [26]. In this study, an inflammatory model of hippocampal neuronal cells was established by LPS to simulate the occurrence of depression which was induced by hippocampal neuronal inflammation. The model showed increased proinflammatory cytokines, abnormal tryptophan metabolism, downregulation of the BDNF pathway, and neuronal injury. So we have measured the levels of the proinflammatory cytokines TNF-*α* and IL-6, the expression of IDO and 5-HT, and the expression of BDNF, NGF, TrkB, TrkA, CREB, p-CREB, and SYP in the hippocampal neuronal cell. Measurement of BrdU/NeuN further confirmed the neuroprotective effects of XYW on depression in the hippocampal neuronal cell inflammation model induced by LPS.

## 2. Materials and Methods

### 2.1. Xiaoyao Pill Quality Control (QC)

The analysis was performed by High-Performance Liquid Chromatography (HPLC) (Thermo, US). The column was C18 column (4.6 × 250 mm, 5 *μ*m), and the chromatographic separation conditions were as follows: column temperature: 30°C; flow rate: 1.0 mL/min; mobile phase: acetonitrile+0.1% phosphoric acid (15 : 85); stock solutions of Xiaoyao Pills were prepared by dissolving 0.4 g of analyte in 25 mL dilute ethanol [21]. The content in Xiaoyao Pills was determined by quantitation paeoniflorin (C_23_H_28_O_11_). Paeoniflorin content should not be less than 4.0 mg in 1.0 g of concentrated pills [11]. The content of paeoniflorin in 1.0 g Xiaoyao Pills is 27.5 mg.

### 2.2. Drugs and Reagents

Xiaoyao Pills (TaiJi, China), LPS (Escherichia coli 055:B5), fluoxetine hydrochloride (FLX), and DAPI were provided by Sigma (St. Louis, MO). Rabbit anti-NeuN antibody (1 : 50; Cat. No. #24307S), mouse anti-BrdU antibody (1 : 1400; Cat. No. #5292S), rabbit anti-TrkB antibody (1 : 1000; Cat. No. #4603), rabbit anti-CREB antibody (1 : 1000; Cat. No. #9197S), rabbit anti-p-CREB antibody (1 : 1000; Cat. No. #9198S), and rabbit anti-*β*-tubulin antibody (1 : 1000; Cat. No. #2128) were all from Cell Signaling Technology. Rabbit anti-synaptophysin polyclonal antibody (1 : 1000; Cat. No. #17785-1-AP) was from Proteintech. Rabbit anti-GAPDH antibody (1 : 1000; Cat. No. #GB11002) was from Servicebio. Goat anti-mouse IgG-FITC (1 : 100; Cat. No. #10) and goat anti-rabbit IgG/Cy3 (1 : 100; Cat. No. #AG04017512) were both from Absin.

### 2.3. Preparation of Xiaoyao Pill Serum

Male Sprague-Dawley rats that weighed 220–240 g were purchased from Chengdu Dashuo. All animals were raised in standard cages in a room of constant temperature and humidity (22 ± 1°C; 40–60%) with a 12 : 12 dark/light cycle (lights on at 8:00 a.m.; off at 8:00 p.m.). The animals were given free access to food and water throughout the experiment. Rats in the XYW group were intragastrically administered XYW (Xiaoyao Pills, 1.86 g·kg^−1^) daily for 14 days; meanwhile, rats in the control groups were intragastrically administered equivoluminal saline daily to ensure isocaloric intake. Rats were anesthetized after 1 h for the last lavage. Then, blood was taken from the abdominal aorta, centrifuged, in a water bath, sterilized with a filter membrane, and stored in a −80°C refrigerator, avoiding repeated freezing and thawing.

### 2.4. Cell Culture and Treatment

Embryonic brains were dissected, and single-cell suspensions of the hippocampus were obtained by mechanical dissociation from Sprague-Dawley rats which were purchased from Chengdu Dashuo. Primary hippocampal neurons were plated at a density of 5 × 10^5^ cells/mL in polylysine-coated culture plates and cultured in DMEM supplemented with 10% FBS at 37°C in a humidified atmosphere of 5% CO_2_/95% air using standard cell culture methods for 24 h. Then, for differentiation of neural stem cells, replace medium with Neurobasal-A medium containing 2% B-27 and 1% L-glutamine. The medium was changed every 3 days. After the 10^th^ day, LPS was added to the medium. Then, 24 h later, hippocampal neurons were incubated with Xiaoyao Pill serum (4% and 8% concentration) in Neurobasal-A medium without 2% B-27 for 48 h. After a preliminary experiment, it was found that Xiaoyao Pill serum concentrations of 4% and 8% had no effect on cell viability.

### 2.5. Cell Identification

**Figure 1:**
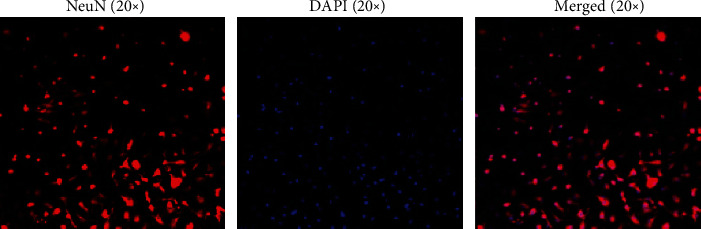
Identification of rat primary hippocampal neurons (20x). The hippocampal neuronal cells were incubated with anti-NeuN antibody (red) and DAPI (blue) and were observed by a laser scanning confocal microscope.

The primary hippocampal neurons were cultured in a laser confocal petri dish for 13 days according to item 2.4. After the completion of culture, abandon the culture medium and add 4% paraformaldehyde to fix for 30 min. Add 0.25% Triton X-100 to permeabilize for 15 min, then incubate with 5% BSA-PBST for 1 h. Cells were incubated overnight after adding rabbit anti-NeuN antibody diluent (1 : 50; Cell Signaling Technology; Cat. No. #24307S), then adding goat anti-rabbit IgG/Cy3 diluent (1 : 100; Absin; Cat. No. #AG04017512), and incubating for 1 h. Images were captured at 20x using a confocal microscope. NeuN is a specific marker for neurons, which can be marked red by NeuN-CY3. After the double staining of NeuN and DAPI, the distribution of red fluorescence and blue fluorescence of the cells cultured for 13 days was basically consistent, indicating that the cultured cells were neurons (Figure 1).

### 2.6. Mechanism Detection

#### 2.6.1. ELISA Analysis for IL-6, TNF-*α*, IDO, 5-HT, BDNF, and *β*-NGF

The levels of IL-6, TNF-*α*, BDNF, and *β*-NGF in cell supernatant and IDO and 5-HT in cell lysate were detected by ELISA, according to the manufacturer's instructions, and the optical density was measured at 450 nm by a microplate reader.

#### 2.6.2. Total RNA Expression in the Hippocampus and Cortex via RT-PCR

The total RNA was used to synthesize cDNA using the FastQuant RT kit (Tiangen, Beijing, China); subsequently, the amplification reactions were carried out in 96-well reaction plates with 20 *μ*L reaction volume (Bio-Rad). The gene primer sequences of *β*-actin, IL-6, TNF-*α*, 5-HT1A, IDO1, BDNF, NGF, TrkB, TrkA, and CREB used in this study are listed in Table 1.

#### 2.6.3. Western Blotting Analysis

RIPA lysate buffer containing 1 mM PMSF was added to each sample to collect the total protein. The total protein concentration of each sample was determined by the BCA method and adjusted all samples to the same concentration. The protein samples were mixed with a 5x loading buffer and denatured at 95°C. The proteins were separated by SDS-PAGE (8% or 15%) and electrophoretically transferred onto polyvinylidene fluoride membranes. The membranes were probed with rabbit anti-TrkB antibody (1 : 1000; Cell Signaling Technology; Cat. No. #4603), rabbit anti-CREB antibody (1 : 1000; Cell Signaling Technology; Cat. No. #9197S), rabbit anti-p-CREB antibody (1 : 1000; Cell Signaling Technology; Cat. No. #9198S), rabbit anti-*β*-tubulin antibody (1 : 1000; Cell Signaling Technology; Cat. No. #2128), rabbit anti-synaptophysin polyclonal antibody (1:1000; Proteintech; Cat. No. #17785-1-AP), and rabbit anti-GAPDH antibody (1 : 1000; Servicebio; Cat. No. #GB11002) overnight at 4°C and then incubated with Anti-rabbit IgG HRP-Linked Antibody (1 : 3000; Servicebio; Cat. No. #GB23303) at 37°C for 1.5 h. Detection was performed using a ChemiDoc XRS^+^ (Bio-Rad, USA) image analysis system.

#### 2.6.4. Immunostaining

Cultured cells were fixed in a PBS solution containing 4% paraformaldehyde for 15 min and washed 3 times with PBS. A PBS solution containing 0.25% Triton was added to the cells for 15 min at room temperature and then incubated with 5% bovine serum albumin for 1 h. After removing this blocking reagent, cells were incubated in a humidified chamber at 4°C overnight with primary antibodies: mouse anti-BrdU antibody (1 : 1400; Cell Signaling Technology; Cat. No. #5292S), rabbit anti-NeuN antibody (1 : 50; Cell Signaling Technology; Cat. No. #24307S), and rabbit anti-NeuN antibody (1 : 50; Cell Signaling Technology; Cat. No. #24307S) diluted in blocking reagent. Then, cells were washed 3 times with PBS and incubated for 1 h in the dark at room temperature in the presence of the fluorescent secondary antibodies: goat anti-mouse IgG-FITC (1 : 100; Absin; Cat. No. #10) and goat anti-rabbit IgG/Cy3 (1 : 100; Absin; Cat. No. #AG04017512). Finally, the coverslips were mounted onto slides in PBS. The preparations were analysed under a fluorescent microscope (Olympus FV1200).

### 2.7. Statistical Analysis

All analyses were performed using SPSS. Data are presented as the mean ± SD. All analyses were performed using one-way ANOVA, *t*-test analysis, or Mann-Whitney test rank-sum analysis. The level of significance was set at *p* ≤ 0.05.

## 3. Results

### 3.1. Xiaoyao Pills Prevent LPS-Induced Inflammation

**Figure 2:**
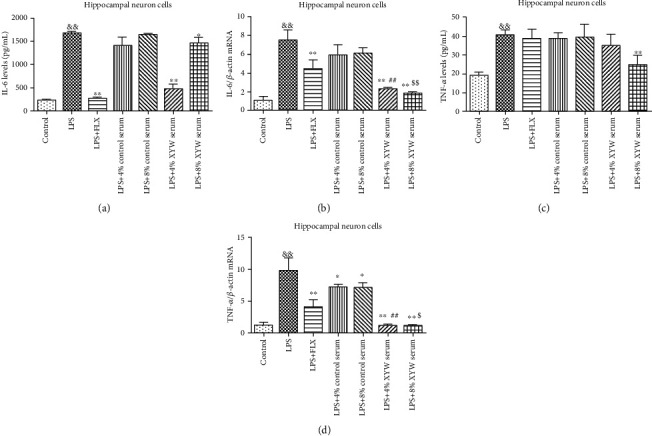
Xiaoyao Pill serum (final concentration of 4% and 8%) reduced the expression of IL-6 and TNF-*α* in hippocampal neuronal cells. The protein levels of IL-6 in supernatant (a). The relative mRNA expression of IL-6 in cell lysate (b). The protein levels of TNF-*α* in supernatant (c). The relative mRNA expression of TNF-*α* in cell lysate (d). The results were expressed as the mean ± SD (*n* = 4‐5). ^&&^*p* < 0.01 compared to the control group. ^∗^*p* < 0.05 and ^∗∗^*p* < 0.01 compared to the LPS group. ^##^*p* < 0.01 compared to the LPS+4% control serum group. ^$$^*p* < 0.01 compared to the LPS+8% control serum group.

As shown in Figures 2(a) and 2(c) and Table 1, after treatment with LPS, inflammatory cytokines, including IL-6 (Figure 1(a), *F* = 2.746, *p* < 0.001) and TNF-*α* (Figure 1(c), *p* = 0.009), are released from hippocampal neuronal cells. Treatment with fluoxetine hydrochloride (FLX) could reduce the IL-6 level in supernatant (Figure 2(a), *F* = 3.291, *p* < 0.001) and downregulate IL-6 mRNA expression in cells (Figure 1(b), *p* = 0.009) and TNF-*α* mRNA expression (Figure 2(b), *F* = 8.377, *p* = 0.001). Treatment with Xiaoyao Pill serum (8%) could decrease the levels of IL-6 (Figure 2(a), 8%: *F* = 4.133, *p* = 0.010) and TNF-*α* (Figure 2(c), 8%: *p* = 0.009) in supernatant, while Xiaoyao Pill serum (4%) also decreased the level of IL-6 in supernatant (Figure 2(a), *F* = 1.262, *p* < 0.001). Furthermore, in cell lysates, Xiaoyao Pill serum (4% and 8%) reduced the expression of IL-6 mRNA (Figure 2(b), 4%: *p* = 0.009; 8%: *p* = 0.009) and TNF-*α* mRNA (Figure 2(b), 4%: *F* = 42.007, *p* < 0.001; 8%: *F* = 47.912, *p* < 0.001). Although the serum of 4% and 8% Xiaoyao Pills could significantly reduce the IL-6 level in the supernatant, there was no obvious dose correlation, which may be related to the complexity of the components of traditional Chinese medicine or the interaction between the components. In conclusion, FLX and the serum of Xiaoyao Pills could inhibit the inflammatory reaction of hippocampal neuronal cells induced by LPS.

### 3.2. Xiaoyao Pills Prevent LPS-Induced Limited 5-HT and IDO

**Figure 3:**
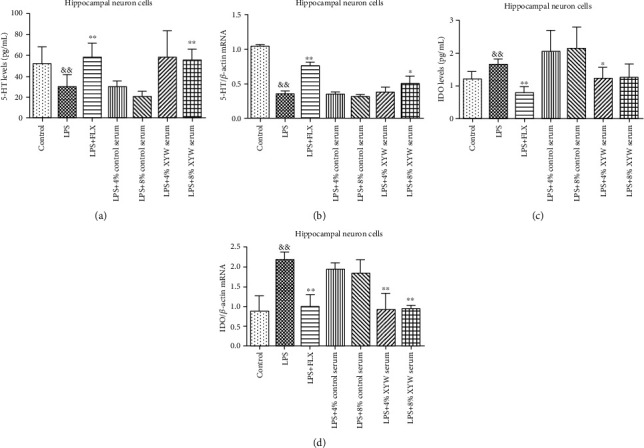
Xiaoyao Pill serum improved the expression of 5-HT and lowered the expression of IDO in hippocampal neuronal cells. The protein levels of 5-HT in cell lysate (a). The relative mRNA expression of 5-HT in cell lysate (b). The protein levels of IDO in cell lysate (c). The relative mRNA expression of IDO in cell lysate (d). The results were expressed as the mean ± SD (*n* = 4‐6). ^&&^*p* < 0.01 compared to the control group. ^∗^*p* < 0.05 and ^∗∗^*p* < 0.01 compared to the LPS group.

We further proved that, in vitro experiments, LPS could increase the protein (Figure 3(c), *F* = 0.709, *p* = 0.006) and its mRNA expression (Figure 3(d), *p* = 0.009) of IDO in hippocampal nerve cell lysate and downregulate the 5-HT level (Figure 3(a), *F* = 0.772, *p* = 0.039) and its mRNA expression (Figure 3(b), *F* = 18.479, *p* < 0.001) compared with the control group. FLX improved the levels of 5-HT (Figure 3(a), *F* = 0.368, *p* = 0.005) and its mRNA (Figure 3(b), *F* = 0.061, *p* < 0.001) in cell lysate and decreased the levels of IDO (Figure 3(c), *F* = 0.081, *p* < 0.001) and its mRNA (Figure 3(d), *p* = 0.009) in cell lysate. Xiaoyao Pill serum (8%) also improved the levels of 5-HT (Figure 3(a), *F* = 0.053, *p* = 0.007) and its mRNA expression (Figure 3(b), *F* = 7.652, *p* = 0.034) in cell lysate, while decreasing the expression of IDO mRNA (Figure 3(d), *p* = 0.009) in cell lysate. Xiaoyao Pill serum (4%) decreased the level of IDO protein (Figure 3(c), *F* = 2.793, *p* = 0.035) and its mRNA expression (Figure 3(d), *p* = 0.009) in cell lysate. These results showed that Xiaoyao Pills could resist the abnormal upregulation of IDO induced by LPS and the abnormal downregulation of 5-HT, thus playing an inhibitory role in the inflammatory response of hippocampal nerve cells.

### 3.3. Xiaoyao Pills Prevent LPS-Induced Reduction of Neurotrophic Factor

**Figure 4:**
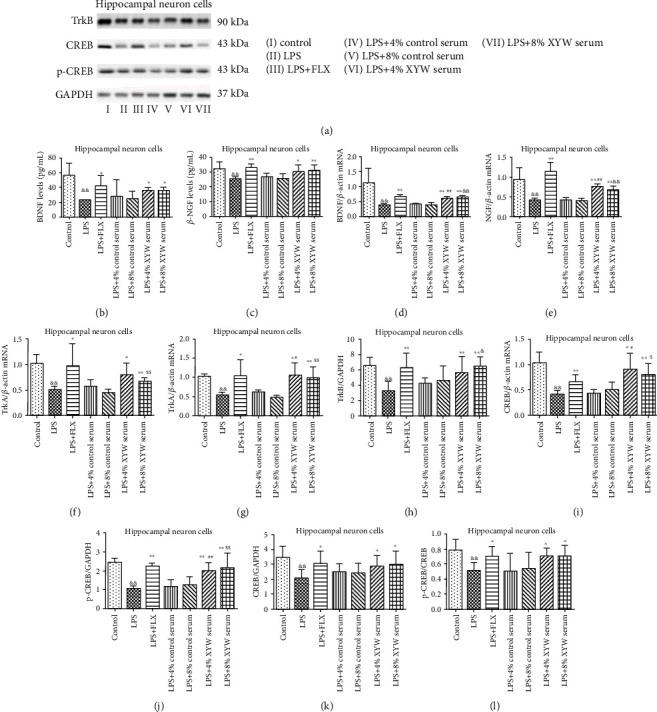
Xiaoyao Pills prevent LPS-induced reduction of neurotrophic factor. Representative blots of relative protein expression of TrkB, CREB, and p-CREB (a). Xiaoyao Pill serum (4% and 8%) improved the levels of BDNF (b) and *β*-NGF (c) in supernatant and increased the transcription level of BDNF (d), NGF (e), TrkB (f), TrkA (g), and CREB (i) and the translation levels of TrkB (h), CREB (k), and p-CREB (j) in cell lysate, as well as the ratio of p-CREB/CREB (l) higher than the LPS group. The results were expressed as the mean ± SD (*n* = 5‐6). ^&&^*p* < 0.01 compared to the control group. ^∗^*p* < 0.05 and ^∗∗^*p* < 0.01 compared to the LPS group. ^##^*p* < 0.05 and ^##^*p* < 0.01 compared to the LPS+4% control serum group. ^$^*p* < 0.01 and ^$$^*p* < 0.01 compared to the LPS+8% control serum group.

In primary hippocampal neuron cells, the production of neurotrophic factors and related factors decreased after LPS treatment in hippocampal neurons, including BDNF (protein expression: Figure 4(b), *F* = 8.856, *p* = 0.008; mRNA expression: Figure 4(d), *F* = 12.023, *p* = 0.024) and NGF (protein: Figure 4(c), *F* = 37.075, *p* = 0.017; mRNA: Figure 4(e), *F* = 14.076, *p* = 0.018), as well as its high-affinity tropomyosin-related kinase, such as TrkB (protein: Figure 4(h), *F* = 4.221, *p* < 0.001; mRNA: Figure 4(f), *F* = 4.157, *p* < 0.001), TrkA (mRNA: Figure 4(g), *F* = 0.010, *p* < 0.001), and CREB (protein: Figure 4(k), *F* = 2.506, *p* = 0.001; mRNA: Figure 4(i), *F* = 5.582, *p* = 0.001), p-CREB (Figure 4(j), *F* = 0.115, *p* < 0.001), and the ratio of p-CREB/CREB (Figure 4(l), *F* = 1.118, *p* = 0.005). Xiaoyao Pill serum (4% and 8% concentrations) and FLX could increase the levels of BDNF (Figure 4(b), FLX: *F* = 4.186, *p* = 0.034; 4% serum: *F* = 8.666, *p* = 0.028; 8% serum: *F* = 5.504, *p* = 0.030) and *β*-NGF (Figure 4(c), FLX: *F* = 3.389, *p* < 0.001; 4% serum: *F* = 5.779, *p* = 0.034; 8% serum: *F* = 3.777, *p* = 0.003) in the supernatant, while increasing the transcription level of BDNF (Figure 4(d), FLX: *F* = 0.890, *p* < 0.001; 4%: *F* = 0.751, *p* < 0.001; 8%: *F* = 0.116, *p* < 0.001), NGF (Figure 4(e), FLX: *F* = 33.082, *p* = 0.002; 4% serum: *F* = 0.231, *p* < 0.001; 8% serum: *F* = 1.288, *p* = 0.001), TrkB (Figure 4(f), FLX: *F* = 4.221, *p* = 0.001; 4% serum: *F* = 8.976, *p* = 0.045; 8% serum: *F* = 0.220, *p* = 0.003), TrkA (Figure 4(g), FLX: *F* = 5.901, *p* = 0.034; 4% serum: *F* = 9.288, *p* = 0.019; 8% serum: *F* = 4.224, *p* = 0.004), and CREB (Figure 4(i), FLX: *F* = 0.537, *p* = 0.004; 4% serum: *F* = 15.732, *p* = 0.024; 8% serum: *F* = 5.745, *p* = 0.006) in the cell lysate, and the protein expression of TrkB (Figure 4(h), FLX: *F* = 4.221, *p* = 0.001; 4% serum: *F* = 4.221, *p* = 0.007; 8% serum: *F* = 4.221, *p* < 0.001), CREB (Figure 4(k), FLX: *F* = 2.506, *p* = 0.021; 4% serum: *F* = 2.506, *p* = 0.049; 8% serum: *F* = 2.506, *p* = 0.033), and p-CREB (Figure 4(j), FLX: *F* = 0.024, *p* < 0.001; 4% serum: *F* = 2.741, *p* < 0.001; 8% serum: *F* = 3.851, *p* = 0.008) in the cell lysate, as well as the ratio of p-CREB/CREB (Figure 4(l), FLX: *F* = 1.291, *p* = 0.024; 4% serum: *F* = 0.079, *p* = 0.011; 8% serum: *F* = 0.053, *p* = 0.021).

The regulation of neurogenesis and synaptic plasticity of neurons is closely related to BDNF. BDNF could self-release from neurons into the extracellular space and bind to TrkB, in turn phosphorylating CREB and playing a neuroprotective role. In this study, we mainly observed the extracellular BDNF level and the protein expression levels of intracellular TrkB, CREB, and p-CREB. The results indicated that Xiaoyao Pills prevented the reduction of neurotrophic factor induced by LPS and activated the BDNF/TrkB/CREB molecular pathway.

### 3.4. Xiaoyao Pills Promote LPS-Damaged Synaptic Growth

**Figure 5:**
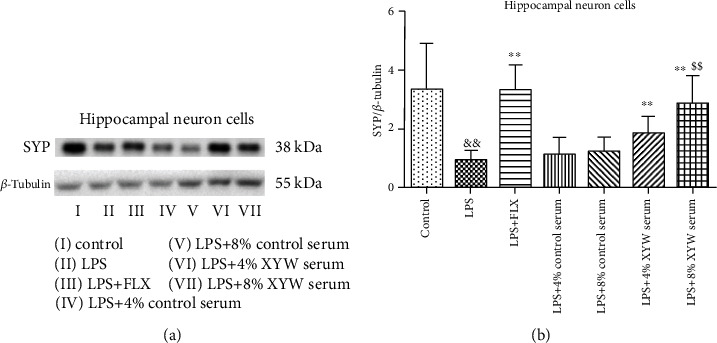
Xiaoyao Pill serum improved the levels of SYP in cell lysates. Representative blots and statistical graphs of relative protein expression of SYP (a, b). The results were expressed as the mean ± SD (*n* = 6). ^&&^*p* < 0.01 compared to the control group. ^∗∗^*p* < 0.01 compared to the LPS group. ^$$^*p* < 0.01 compared to the LPS+8% control serum group.

Synaptophysin (SYP) is widely regarded as scaffold proteins, involved in the regulation of synaptic function. The level of SYP in neuron cells could reflect whether neuronal synapses are damaged. In hippocampal neuron cells, LPS induced synaptic dysfunction (Figure 5(b), *F* = 9.506, *p* = 0.011). Xiaoyao Pill serum and FLX ameliorated synaptic dysfunction by promoting the expression of synaptophysin (Figure 5(b), FLX: *F* = 4.430, *p* < 0.001; 4% serum: *F* = 1.106, *p* = 0.006; 8% serum: *F* = 7.353, *p* = 0.003), suggesting Xiaoyao Pills could promote synaptic growth damaged by LPS.

### 3.5. Xiaoyao Pills Prevent the LPS-Induced Decrease in the Proliferation of Hippocampal Neurons

**Figure 6:**
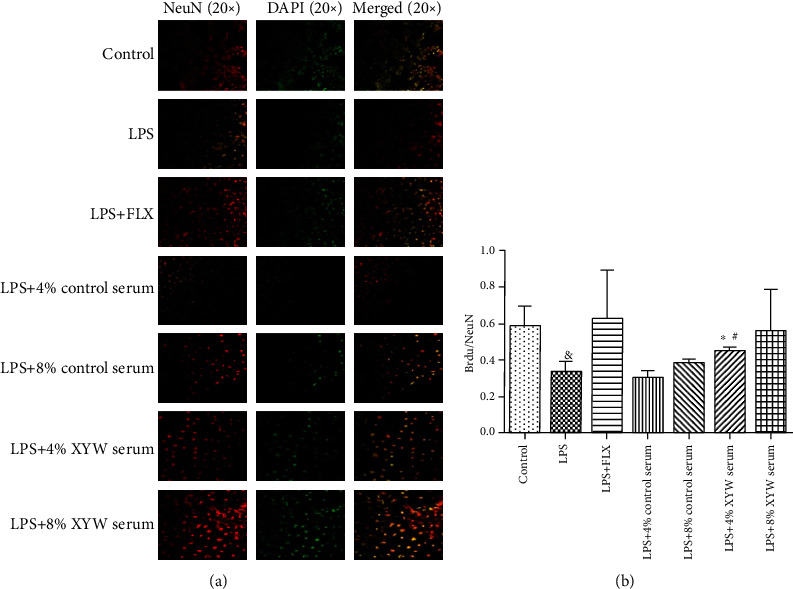
Xiaoyao Pills promote LPS-damaged synaptic growth. The hippocampal neuronal cells were incubated with anti-NeuN antibody (red) and anti-BrdU antibody (green) and were observed by a laser scanning confocal microscope (20x) (a). The statistical graphs of the ratio of BrdU's average optical density/NeuN's average optical density (b). Data were the mean ± SD (*n* = 4). ^&^*p* < 0.05 compared to the control group. ^∗^*p* < 0.05 compared to the LPS group. ^#^*p* < 0.05 compared to the LPS+4% control serum group.

BrdU, also known as deoxyuridine bromide, is a synthetic thymidine analogue that substitutes thymine nucleosides for selective binding to cellular DNA during the S phase. Thus, cell DNA synthesis and cell division and apoptosis were detected. BrdU is often used as a marker of cell proliferation. BrdU immunofluorescence chemistry has been used to study the development of the nervous system and to identify neurogenesis in the brain. The average optical density of BrdU/NeuN double-labelled positive cells in the model group was significantly lower than that in the control group (Figure 6(b), *F* = 6.939, *p* = 0.013), and the ratio of BrdU/NeuN was significantly higher in the Xiaoyao Pill serum (4%) group than that in the LPS group (Figure 6(b), *F* = 7.754, *p* = 0.019). It indicated that Xiaoyao Pills could prevent the decrease in the proliferation of hippocampal neurons induced by LPS.

## 4. Discussion

Neurons are the basic structural and functional units of the nervous system; the role of neurons is to integrate and transmit signals. And the changes of neuronal structure and function in the brain in response to various stimuli, including stress and inflammation, cause nerve damage and eventually lead to depression. Depression is one of the most common mental illnesses. It is a multifactorial disease with both genetic and environmental factors contributing to its pathogenesis. And it affects multiple behavioral areas and presents a variety of symptoms, namely, depressed mood, anhedonia, anxiety, and cognitive impairments, leading to severe disability and impaired quality of life in patients.

Neuroinflammation is considered to be an important pathological cause of depression. It is related to the cytokine hypothesis; patients with major depression have been found to display enhancive inflammatory biomarkers, including inflammatory cytokines. And activation of the inflammatory pathway in the brain reduces neurotrophic support and altered glutamate release/reuptake, as well as oxidative stress, leading to excitotoxicity, consistent with neuropathological findings of depression characteristics [27]. The proinflammatory cytokines can cause sickness behavior and cellular damage [28, 29]. An increase of inflammation can lead to negative emotion, which was also supported by studies that induced inflammation through the injection of LPS. Stimulated increases in proinflammatory cytokines such IL-1*β*, IL-6, and TNF-*α* were associated with depressive symptoms, and proinflammatory cytokines have been recently shown to interact with the brain, affecting neurotransmission, neuroendocrine activity, and brain structure and function, thereby changing emotion, cognition, and behavior. One of the molecular mechanisms that can contact the inflammation with emotional cognition is the proinflammatory cytokine effect on the serotonergic system. Proinflammatory cytokines activate IDO, an enzyme involved in the synthesis of kynurenine from tryptophan. Central and peripheral activation of IDO causes increased catabolism of tryptophan, leading to 5-HT deficiency and neurotoxic metabolite production. Depressive-like behaviors are associated with the reduced synthesis of 5-HT and the production of neurotoxic metabolites in the limbic-cortical-striatal-pallidal-thalamic (LCSPT) circuit [30]. Our study revealed the hippocampal neuron contents of IL-6. TNF-*α* and IDO in the LPS group were significantly higher than those in the control group. And the levels of 5-HT in the LPS group were significantly less than those in the control group. Xiaoyao Pills and FLX inhibited the increase of IL-6, TNF-*α*, and IDO levels in hippocampal neurons induced by LPS and the decrease of 5-HT levels in hippocampal neurons, showing a protective effect on the damage of model cells.

Additionally, there is abundant evidence that depressed patients are associated with the reduction of hippocampal volume, neuronal atrophy, and neuronal loss. The cause may be the lack of neurotrophic factors [31, 32]. LPS can induce neuronal death, decrease neurogenesis, and impair synaptic plasticity and memory. Studies have shown that LPS influenced neurotrophin levels in the brain; the neuroprotection mediated by neurotrophins is compromised by systemic immune activation induced by LPS [33]. BDNF belongs to the family of nerve growth factors and plays an important role in neuronal development, including growth, differentiation, and survival. Preclinical studies have shown that exposure to stress causes hippocampal atrophy and cell loss, as well as decreased neurotrophic/growth factor expression. Therefore, it supports the neurotrophic/neurogenic hypothesis of depression and antidepressant effects [34]. BDNF exerts its neurotrophic effects by activating the TrkB [35]; the phosphorylation of TrkB can activate the transcription factor CREB's gene expression and then play an antidepressant effect [36], promote neuronal survival [37], and strengthen synaptic plasticity [38]. BDNF is abundantly expressed in the brain and plays a role in maintaining the structure of adult brain cells [39]. And in rodents, direct infusion of BDNF in the hippocampus showed antidepressant-like effects by increased levels of TrkB, ERK, CREB, and phosphorylated ERK [40]. NGF is a growth factor first described as a neurite outgrowth factor [41]. And it was involved in the survival of neurons and the proliferation of neural stem cells in rats [42]. BDNF and NGF have also been shown to represent an important factor in the regulation of neurogenesis and synaptic plasticity [43]. In this study, we observed that the expression levels of NGF, BDNF, TrkA, TrkB, CREB, p-CREB, and p-CREB/CREB in hippocampal neurons of the LPS group were significantly decreased, while Xiaoyao Pills could improve the expression levels of the above indicators. The results suggest that the antidepressant effect of Xiaoyao Pills may be related to activating the NGF/BDNF-TrkA/TrkB-CREB pathway.

The BDNF signaling pathway regulates synaptic plasticity in the hippocampus, and BDNF infusion of rat hippocampus induces LTP and triggers synaptic enhancement [44]. As demonstrated by previous studies, BDNF plays a critical role in the action of antidepressants through neuronal plasticity. Synaptic plasticity represents one of the most important functions of the brain, including the ability to collect, evaluate, and store information. This function is associated with depression, including loss of neurotrophic factor support and elevation of inflammatory cytokine. Synaptophysin is now widely accepted as scaffold proteins, which are involved in the regulation of synaptic function. In our present study, LPS induces a decrease in synaptic protein expression in hippocampal neurons that the treatment of Xiaoyao Pills significantly ameliorated synaptic protein reduction.

Neurogenesis has been widely described as a key function of the hippocampus. The reduction of neurogenesis increases innate anxiety-like and approach-avoidance behavior [45]. Most animal studies have found that hippocampal neurogenesis could be achieved by directly targeting the HPA axis and related neuropeptides, thereby regulating depressive-like behaviors by promoting neurogenesis [46]. Increasing adult hippocampal neurogenesis is sufficient to reduce anxiety and depression-like behaviors [47]. In clinical research, patients with depression also exhibit decreased levels of neurogenesis [48]. Therefore, increased neurogenesis may be a potential therapeutic strategy for treating depression. To elucidate whether the impairment of hippocampal neurogenesis was related to depression, we quantified BrdU-positive cell numbers in hippocampal neuron cells and observed that infection of LPS significantly decreased survival of newborn cells and immature neurons which were consistent with previous reports. The serum of Xiaoyao Pills has a protective effect on LPS-induced damage of newborn cells and immature neurons.

Our previous studies have shown that Xiaoyao Pills administered intragastrically could reduce the levels of cytokines and mediators related to inflammation, enhance the expression of neurotrophic factors and synaptic proteins, and improve nerve injury, so as to exert the inhibitory effect on behavioral abnormalities in depression-like model rats induced by lipopolysaccharide [26]. In this study, the primary hippocampal neurons of rats were used as the experimental carrier, and the Xiaoyao Pill-containing serum of rats was used as the observation subject to further study the effect of Xiaoyao Pills on the inflammatory response of hippocampal neurons *in vitro*.

In this study, the serum concentrations of Xiaoyao Pills selected were 4% and 8%, and the above concentrations did not affect the cell activity and had some pharmacological effects. In the experiment, there was no significant dose-response relationship between the two concentrations, which may be related to the complexity of Chinese herbal compound components or the interaction between components [49], which needs to be further explored in combination with the research work of serum drug chemistry. In addition, traditional Chinese medicine compounds are suitable for individualized treatment plans for different body conditions, and the therapeutic effects are not judged by dosage [50, 51]. However, the efficacy of the two concentrations of the serum containing Xiaoyao Pills was similar, at least suggesting that Xiaoyao Pills may alleviate depressive symptoms by suppressing the inflammatory response or activating the NGF/BDNF-TRKA/TrkB-CREB pathway after entry into the body.

**Figure 7:**
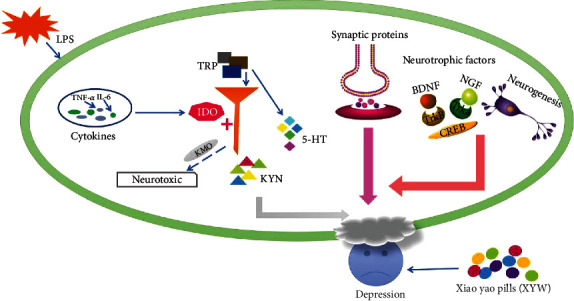
Graphical abstract. Xiaoyao Pills (XYW) ameliorated the depression-like behavior, decreased the levels of inflammatory indicators, increased those of neurotrophic factors and synaptic proteins, and increased hippocampal neurogenesis.

In conclusion, this study showed that LPS could increase the level of proinflammatory cytokines in neuronal cells, thereby increasing the IDO level, promoting tryptophan metabolism to kynurenine resulting in a reduction in 5-HT levels. In addition, LPS could induce the decrease of synaptic protein level in neuron cells, as well as block BDNF and NGF pathways, then inhibit the hippocampal neurogenesis. All the above pathological mechanisms are closely related to the occurrence of depression [52–54] (Figure 7). Pretreatment with Xiaoyao Pills significantly reduced the level of cytokines and mediators related to inflammation, increased the expression of neurotrophic factors and synaptic proteins, and alleviated nerve injury. These findings suggest that Xiaoyao Pills could play a neuroprotective role by inhibiting the neuroinflammatory response, promote the recovery of injured nerves, and thus reduce the symptoms of depression (Figure 7), providing possible treatment basis for its clinical application for depression.

## Figures and Tables

**Table 1:** Gene primer sequence.

Gene	Primer	Primer sequence (5′ to 3′)	Product size (bp)
*β*-Actin	Forward primer	CACCCGCGAGTACAACCTTC	207
	Reverse primer	CCCATACCCACCATCACACC	
IL-6	Forward primer	AGAGACTTCCAGCCAGTTGC	115
	Reverse primer	CTGGTCTGTTGTGGGTGGTA	
TNF-*α*	Forward primer	GATCGGTCCCAACAAGGAGG	138
	Reverse primer	GCTTGGTGGTTTGCTACGAC	
5-HT1A	Forward primer	TGATCTCGCTCACTTGGCTC	145
	Reverse primer	AAAGCGCCGAAAGTGGAGTA	
IDO1	Forward primer	GCATCAAGACCCGAAAGCAC	154
	Reverse primer	GTTGCCCTTCCAACCAGACA	
BDNF	Forward primer	TAGGCAGAATGAGCAATGTC	178
	Reverse primer	CCCAAGAGGTAAAGTGTAGAAG	
NGF	Forward primer	TGGAGATAAGACCACAGCCA	197
	Reverse primer	TGACAAAGGTGTGAGTCGTG	
TrkB	Forward primer	TGCTCAAGTTGGCGAGACAT	151
	Reverse primer	GTCCCAGGAGTTCAGCTCAC	
TrkA	Forward primer	CCCTCCTGATGTCTACGCCA	139
	Reverse primer	CTCCTAGCCCAGAACGTCCA	
CREB	Forward primer	AGCCGGGTACTACCATTC	244
	Reverse primer	GCTGCTTCCCTGTTCTTC	

## Data Availability

The datasets generated and/or analysed during the current study are available from the corresponding author on reasonable request.

## Abbreviations

XYW: Xiaoyao Pills
FLX: Fluoxetine hydrochloride
LPS: Lipopolysaccharide
IL-6: Interleukin 6
TNF-*α*: Tumour necrosis factor alpha
IDO: Indoleamine 2,3-dioxygenase
5-HT: 5-Hydroxytryptamine
BDNF: Brain-derived neurotrophic factor
NGF: Nerve growth factor
TrkB: Tropomyosin receptor kinase B
TrkA: Tropomyosin receptor kinase A
CREB: cAMP response element-binding protein
PSD95: Postsynaptic density protein 95
SYP: Synaptophysin
BrdU: 5-Bromo-2-deoxyUridine
NeuN: Vertebrate neuron-specific nuclear protein.
